# Design and Development of Non-Contact Bio-Potential Electrodes for Pervasive Health Monitoring Applications

**DOI:** 10.3390/bios7010002

**Published:** 2017-01-01

**Authors:** Anthony J. Portelli, Slawomir J. Nasuto

**Affiliations:** School of Biological Sciences, University of Reading, Whiteknights Campus, RG6 6UA, UK; anthonyportelli2@hotmail.com

**Keywords:** capacitive, electrodes, wireless, non-contact

## Abstract

For the advent of pervasive bio-potential monitoring, it will be necessary to utilize a combination of cheap, quick to apply, low-noise electrodes and compact electronics with wireless technologies. Once available, all electrical activity resulting from the processes of the human body could be actively and constantly monitored without the need for cumbersome application and maintenance. This could significantly improve the early diagnosis of a range of different conditions in high-risk individuals, opening the possibility for new treatments and interventions as conditions develop. This paper presents the design and implementation of compact, non-contact capacitive bio-potential electrodes utilising a low impedance current-to-voltage configuration and a bootstrapped voltage follower, demonstrating results applicable to research applications for capacitive electrocardiography and capacitive electromyography. The presented electrodes use few components, have a small surface area and are capable of acquiring a range of bio-potential signals.

## 1. Introduction

The detection and analysis of biological electrical signals (bio-potentials) from the surface of the skin, has shown to be a powerful tool for the diagnosis of clinical conditions, in addition to applications in prosthetic limb control and Brain Computer Interfacing (BCI).

The primary clinical application of bio-potential acquisition systems has remained largely the same for the past 70+ years i.e., wet resistive electrodes applied directly to the surface of the skin [1]. This wet resistive technology suffers from several drawbacks which limit the practical deployment of such systems into effective, casual and consumer driven, non-research oriented markets. Wet electrode application is difficult and time-consuming due to the presence of the required electrolytic gel. It can also be painful, as it is often necessary to use an abrasive process to remove grease and particles from the skin prior to electrode placement to achieve desired low impedances [2]. The gel can also diffuse through the subject’s hair causing nearby sensors to short (limiting the effective spatial resolution) and will eventually dry out, thus altering the impedances, requiring re-application.

Demonstrated 40 years ago, dry resistive electrodes [3] have become more commonly reported and commercial systems based on this technology have been released (e.g., the “Sahara” dry electrodes from g.Tec [4]). Dry electrodes do not require electrolytic gel as they incorporate impedance transforming electronics next to the measurement site (the skin-electrode interface). This electronic device typically converts voltage from the high impedance skin interface into a low impedance output to maximise the transfer of voltage into amplification circuitry. Without the wet interface however, these electrodes are more susceptible to skin irregularities (sweat, grease, hair etc.) and motion artifacts. Dry electrodes also suffer from the necessity for constant uniform electrical contact, any displacement away from the surface of the skin or friction against the skin will cause large disruptive artifacts [5,6].

Capacitive electrodes are a more recent development of dry electrode technology. By insulating the detection disc of the sensor, it is possible to consider the interface in the same way as a parallel plate capacitor [7]. As with dry resistive electrodes, capacitive electrodes are highly sensitive to artifacts caused by movement and environmental noise [8].

Special techniques such as neutralisation and guarding [9] are able to mitigate some of these issues but involve placing an increasing number of components on the electrode backing circuitry. This, in turn, increases the overall size and cost of the sensor [10,11].

An increase in sensor detection disc size is also required due to the weak coupling (capacitive) of the electrode to the body under measurement. In comparison to resistive electrodes, high signal attenuation is observed in capacitive electrodes. Attempts to increase the coupling (thereby increasing the signal to noise ratio) include increasing the size of the detection disc to approximately double the diameter of typical wet electrodes (from 9–12 mm to 25 mm).

Despite these issues, capacitive electrodes have been shown to be capable of acquiring bio-potentials signals that rival that of traditional electrode technologies [12].

In contrast to the developments made in acquisition technology, the amplification of the signals is still achieved using traditional methods. Differential amplifiers, instrumentation amplifiers [13], as well as driven right leg (DRL) circuits, are commonplace [14].

Most electrode circuits developed for both active resistive and capacitive electrodes utilise a high impedance input interface which is then transformed into a low impedance output by a voltage follower or buffer amplifier. The electrodes presented here rely on an inverting configuration and a low input bias current single operational amplifier, combined with a modified Printed Circuit Board (PCB) shielded base as shown in [10].

Expanding on previous work in [15], we demonstrate that a low impedance high sensitivity current-to-voltage converter for active electrodes is able to be used in a non-contact (capacitive) mode to detect Electrocardiography (ECG) and Electromyography (EMG) signals. In this paper, we show that the low impedance input electrodes can perform at least as well as the high impedance capacitive electrode counterpart and compare the results obtained against standard Ag/AgCl electrodes.

## 2. Materials and Methods

Research into electronic hardware supporting bio-potential signal acquisition concentrates on identifying operational amplifiers that, when combined with the correct supporting passive components, are able to buffer the signals acquired from the body whilst introducing a minimum of noise. The most prominent examples found in current literature are the OPA129 operational and INA116 instrumentation amplifiers [10,12,16,17]. These are characterised by low bias current requirements, low current noise and low input referred noise. It is an important requirement that amplifiers chosen to support active (especially capacitive) electrodes have a low input bias current requirement, as this helps to minimise voltage error in the output. However, this current can also saturate the amplifier if a purely capacitive input is used. This is usually countered by use of biasing circuitry which grounds the accumulation of these currents. In this paper, we focus on the use of the LMP7721 Texas Instruments (TI) operational amplifier (OPAMP) that has the same low input bias current characteristics as the INA116 but in a single OPAMP package and an inverting configuration with a lower practical input impedance.

It has been shown that active resistive and capacitive electrodes can be built directly onto printed circuit boards (PCBs) [10,14,18] using the bottom copper plane as the detection disc. This is a favourable design as it contains the necessary electrode electronics as close to the measurement site as possible, reducing the possibility of extraneously induced noise, minimising the size and allowing for flexibility of size and reproducibility. For capacitive electrodes this is complementary as, with appropriate design, environmental shielding can be built into the electrode. A variation of this technique was used in the construction of the presented technology.

Due to the high impedance skin-electrode interface, dry electrodes (capacitive and resistive) can be very sensitive to environmental noise, therefore it is beneficial to use some method of shielding i.e., a grounded box surrounding the electrode and supporting circuitry. However, the operation of capacitive electrodes requires a driven shield or guard surrounding the detection disc [19]. This provides relief from parasitic capacitance effects (e.g., capacitive signal division) and will increase immunity against environmental noise. Surrounding the entire electrode is largely unnecessary assuming appropriate circuit layout techniques, as most environmental noise is detected from the subject under measurement.

**Figure 1:**
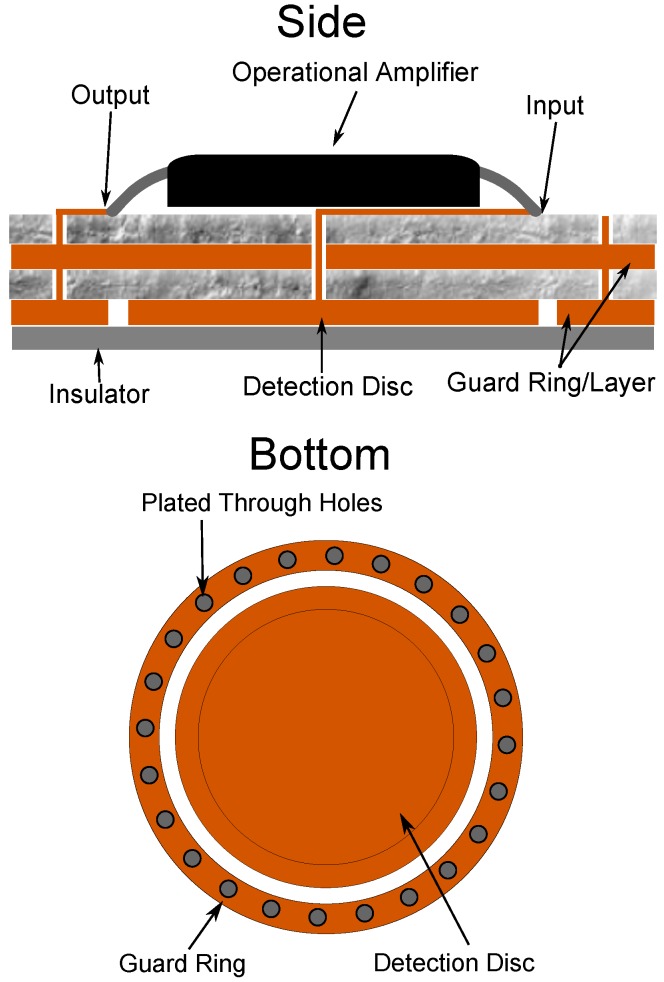
Diagram of the layers of the basic design of a capacitive sensor on a PCB.

To achieve this guarding, an outer ring and inner plane of the printed circuit board were connected to the output of the impedance transformation buffer. An evenly spaced ring of plated through holes was placed around the guard ring, constructing a complete Faraday cage, illustrated in Figure 1. This base was constructed using a four layer PCB.

The following describes the design and implementation of the high sensitivity current-to-voltage converter electrode and the high impedance counterpart electrode designed for comparison.

### 2.1. Low Impedance Design Circuitry

There appears to exist a dichotomy between ensuring low impedance contact between the skin with passive electrodes and attempting, through circuitry, to maximise the interface impedance between the skin when designing active electrodes.

To address this, these electrodes were considered as an alternative to high impedance configurations. This provides lower power consumption, better stability, natural neutralisation of amplifier common-mode and difference-mode capacitance [20] as well as better noise performance through use of carefully selected, off-the-shelf amplifiers.

**Figure 2:**
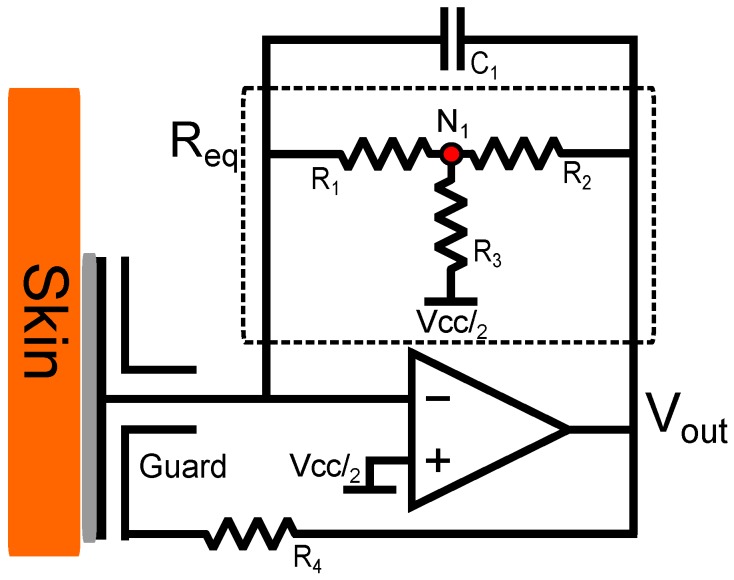
Schematic of the low impedance capacitive sensor design with highlighted T-network $R_{eq}$.

A trans-impedance (current-to-voltage converter) amplifier configuration requires an extremely large feedback resistor to amplify small currents. Single high impedance resistors are expensive, difficult to obtain, can cause noise to be induced into the circuit through Johnson–Nyquist noise and have very large tolerances (≈20+%). However, this problem was mitigated using a T-network which replaces a single resistor with three lower impedance resistors equating to equivalent impedance through application of Kirchoff’s current law. The complete electrode design is shown in Figure 2.

The addition of a capacitor C1 to the feedback loop prevents oscillation and aids in stability of the circuit [21] whilst limiting the bandwidth.

The output voltage of a trans-impedance amplifier configuration with a single resistor in the feedback loop is expressed as:

(1) $$V_{out}=-R_{eq}I_{i}$$

The simplified s-domain transfer function from the circuit shown in Figure 2:

(2) $$G\left(s\right)=\frac{-R_{eq}}{1+sC_{1}R_{eq}}$$

Through the summation of elements at the resistor junction $N_{1}$ in Figure 2:

(3) $$\frac{\left(0-V_{x}\right)}{R_{1}}+\frac{\left(0-V_{x}\right)}{R_{3}}=\frac{\left(V_{x}-V_{0}\right)}{R_{2}}$$

Leading to the equivalent resistance:

(4) $$R_{eq}=R_{1}\left(1+\frac{R_{2}}{R_{1}}+\frac{R_{2}}{R_{3}}\right)$$

Using Equation (4) an equivalent resistance can be calculated for 1 TΩ as:

(5) $$R_{1}=R_{2}=5\;G\Omega ,R_{3}=25.25\;M\Omega$$

(6) $$G\left(s\right)=\frac{-R_{1}\left(1+\frac{R_{2}}{R_{1}}+\frac{R_{2}}{R_{3}}\right)}{1+sC_{1}\left(R_{1}\left(1+\frac{R_{2}}{R_{1}}+\frac{R_{2}}{R_{3}}\right)\right)}$$

Developed through experimental design, the best results were achieved when resistors $R_{1}$ and $R_{2}$ were balanced. The transfer function of the electrode is shown in Equation (6) and expanded in Equation (7).

(7) $$G\left(s\right)=\frac{\left(-R_{2}R_{3}-R_{1}R_{3}-R_{1}R_{2}\right)}{R_{3}+s\left(C_{1}R_{2}R_{3}+C_{1}R_{1}R_{3}+C_{1}R_{1}R_{2}\right)}$$

Based on these equations, with an equivalent feedback resistance of 1TΩ the circuit should yield 1 V/pA sensitivity.

The 5GΩ resistors are readily available in small surface mount packages. However, 25.25MΩ resistors are not, therefore 22MΩ resistors were used. From Equation (5), the use of $R_{3}=22\;M\Omega$ and $R_{1}=R_{2}=5\;G\Omega$ creates an equivalent 1.145TΩ resistance, providing an approximate sensitivity of 1.145 V/pA.

In theory, usage of a T-network with such a high equivalent resistance would only serve to increase noise and lower the achievable signal to noise ratio. The input bias current of the amplifier can contribute to voltage offset errors. The utilised OPAMP states a 0.01 pA/$\sqrt{Hz}$ input referred current noise as well as a 3 fA input bias current. Therefore, bias current errors are less of a concern and are further mitigated by the use of this T-network. This produces a lower input impedance stable amplifier configuration capable of detecting bio-potential signals from the body without contact.

A 100Ω resistor is used to couple the output of the OPAMP to the guard ring and guard plane, $R_{4}$ which has the effect of guarding the input disc. As the configuration is inverting and no electrical contact is used, this acts to add a further parallel capacitive term between the output and input, aiding stabilisation.

### 2.2. High Impedance Bootstrapped Circuitry

The bootstrapped design here is presented as a high-impedance counterpart to the proposed current-to-voltage configuration for comparison. As demonstrated in [12], a basic rail-to-rail operational amplifier can be utilised as an impedance transformer for capacitive electrodes; this configuration produces an electrode with an undefinable direct current (DC) operating point as the capacitive leakage current charges the input. This undefinable offset can cause difficulties when differentially amplifying bio-potential signals as the offset voltage between the two electrodes used in the differential amplification is also amplified causing amplifier saturation. This can be overcome using high-pass filters on the output of the electrode but this can degrade the common-mode rejection ratio (CMRR) of differential and instrumentation amplifiers. This issue is solved in this design by introducing a resistor network that achieves biasing and a stable mid-rail operating point.

**Figure 3:**
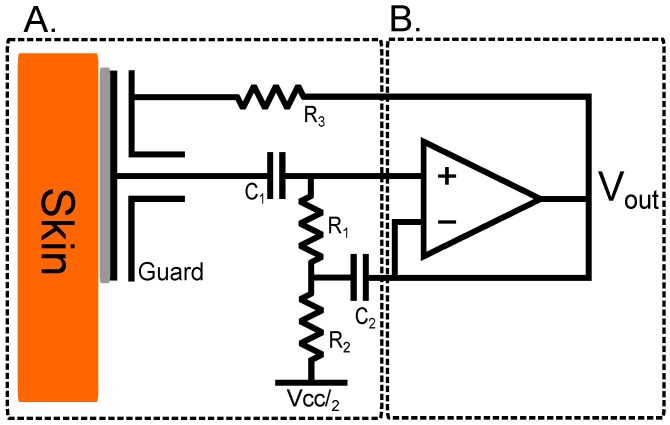
Schematic of the high impedance design configuration showing bootstrapped front end (**A**); and voltage follower (**B**).

In this implementation, the LMP7721 amplifier is configured as an impedance transforming buffer and is coupled with a bootstrapping network. This network provides a stable DC operating point within the supply rails which overcomes DC drift, and prevents saturation of the inputs due to leakage currents. Incorporating positive feedback into the non-inverting terminal of the LMP7721 serves to increase the apparent input impedance of the amplifier; the full design is shown in Figure 3. The output of the buffer is fed back into the inverting terminal of the operational amplifier, keeping the voltages at the inverting and non-inverting terminals equal.

To overcome effects of input bias current saturation, the resistor network allows a path for currents to flow to ground. The addition of $R_{1}$ and $R_{2}$ alone would create a potential division between the ground and the non-inverting terminal of the operational amplifier and, due to the fact that the resistor network has a much smaller impedance than that of the operational amplifier input, much of the signal would be lost. The full bootstrapping network shown in Figure 3A incorporates positive feedback from the output of the buffer amplifier into its non-inverting terminal through the RC network formed by $R_{1}$ and $C_{2}$, countering the potential division. At frequencies above the RC network cut-off frequency, no voltage drop is observed across resistor $R_{1}$. Therefore, no current flows through it and so the observed impedance tends towards infinity, in the ideal case.

The buffer configuration acts as a voltage source with a very low output impedance; when sending the acquired signal on to additional circuitry with a high input impedance, this increases the signal’s immunity to induced environmental noise.

The s-domain transfer function is shown in Equation (8).

(8) $$G\left(s\right)=\frac{\left(C_{1}R_{2}+C_{1}R_{1}\right)s+\left(C_{1}C_{2}R_{1}R_{2}\right)s^{2}}{1+\left(C_{1}R_{2}+C_{1}R_{1}\right)s+\left(C_{1}C_{2}R_{1}R_{2}\right)s^{2}}$$

The values chosen for $R_{1},R_{2}$ and $C_{1}$ and $C_{2}$ were selected specifically to maintain wide frequency characteristics without compromising low current noise. $R_{1}$ and $R_{2}$ were kept the same at 50GΩ and $C_{1}$, 10 nF and $C_{2}$ at 10 μF.

$R_{3}$ is a 100Ω resistor used to couple the output of the operational amplifier to the guard ring and guard plane of the electrode [20] aiding in environmental noise rejection.

## 3. System Configuration

### 3.1. Electrode Configuration

**Figure 4:**
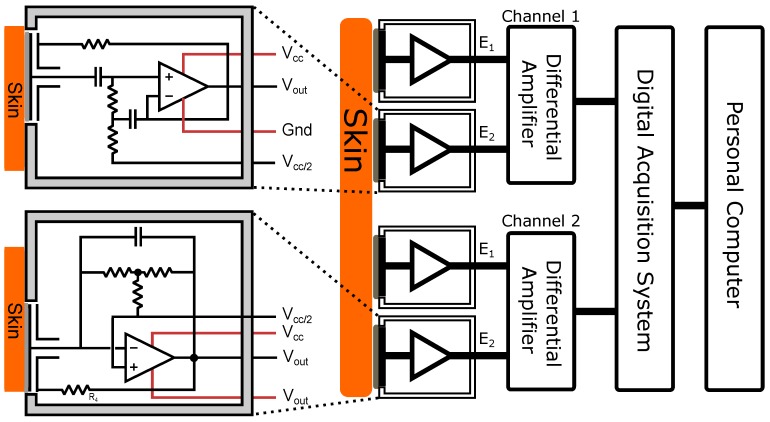
System configuration.

For the acquisition of the data presented in the results section, signals from both AgCl electrodes and the developed electrode designs were sent through separate channels of the digital acquisition system. Each channel represented the amplified differential output of a pair of electrodes. These differential measurements were taken when each pair of electrodes was placed as close to its counterpart as possible; i.e., with respect to Figure 4, Channel 1 $E_{1}$ and Channel 2 $E_{1}$ were put as geographically close to each other as were Channel 1 $E_{2}$ and Channel 2 $E_{2}$. Where a contact ground was used, this was placed in a non-relevant portion of the anatomy. A high-level system view is shown in Figure 4.

The designs detailed in the previous sections utilise a 3.3 V system voltage which powers the electrodes, amplification circuitry and digital acquisition circuitry. The supply is generated from a Lithium Polymer battery cell which is regulated to 3.3 V. The zero point reference was half of the supply voltage, 1.625 V.

The input of the amplification circuitry was universal to both active and passive electrodes and utilised high-pass filtering on its inputs.

### 3.2. Analogue to Digital Conversion and Data Acquisition

Data acquisition is achieved through the use of a Microchip ds33EP (PIC) series micro controller. The PIC acquires the data from the analogue-to-digital (A/D) converter chips, and packages the data to be sent via wireless interface to a PC.

A/D conversion for the data acquisition system (DAQ) is achieved through the use of a Texas Instrument ADS8326 16-bit single channel A/D converter. The digital chip interface connects directly with the PIC serial peripheral interface (SPI). It is possible to acquire digitized data from the chip up to 250 thousand samples per second (250 Ksps) making it suitable for the conversion of biological signals. For testing purposes, the sample rate used was 500 Hz in all tests with a second-order Butterworth anti-aliasing filter with a corner frequency of 200 Hz.

Use of a wireless interface in conjunction with the insulated capacitive electrodes makes the system intrinsically safe as, even in the presence of a contact ground, there is no path for current to flow through the subject being measured.

## 4. Results

The results from the characterisation of the current-to-voltage converter electrode versus the high impedance capacitive electrode and Ag/AgCl conductive electrodes are shown. Each measurement was taken utilising a pair of electrodes in a differential measurement configuration and data wirelessly transmitted to a Personal Computer (PC) for offline analysis.

Comparison is made with a system gain of 250 (0.1% tolerance resistors used) on all channels. The results presented were obtained in an unshielded “noisy” lab room with multiple PCs and other line powered equipment operating in it. The results detail a benchmark comparison of both designs of electrodes compared against Ag/AgCl electrodes and the acquisition of bio-potentials from human subjects.

All experiments were conducted by placing wet electrodes on the desired measurement sites and placing the required capacitive electrodes as close as physically possible. After amplification and anti-alias filtering, the output of both amplifiers was fed into the same digitisation circuitry so that the samples could be compared without bias.

### 4.1. Benchmarking

The Bode100 measurement device from Omicron labs was used for characteristic identification of the developed electrodes; gain/phase response and the gain/phase relationship with respect to distance from source. This was achieved by using a two-port network approach; the Bode100 generates a sine wave at incrementing frequencies (the source), and then differentially measures between two channels. Channel one was connected to the generated waveform and the second channel was connected to the output of the capacitive electrode being measured. The source (connected to a probe for channel 1) was separated from the capacitive electrode detection disc by a 0.11 mm strip of dried cellulose. For measurement of the Ag/AgCl electrodes, the source was connected directly through the electrolytic gel. The result is the difference between the power and phase of the source signal as compared with the resultant signal.

**Figure 5:**
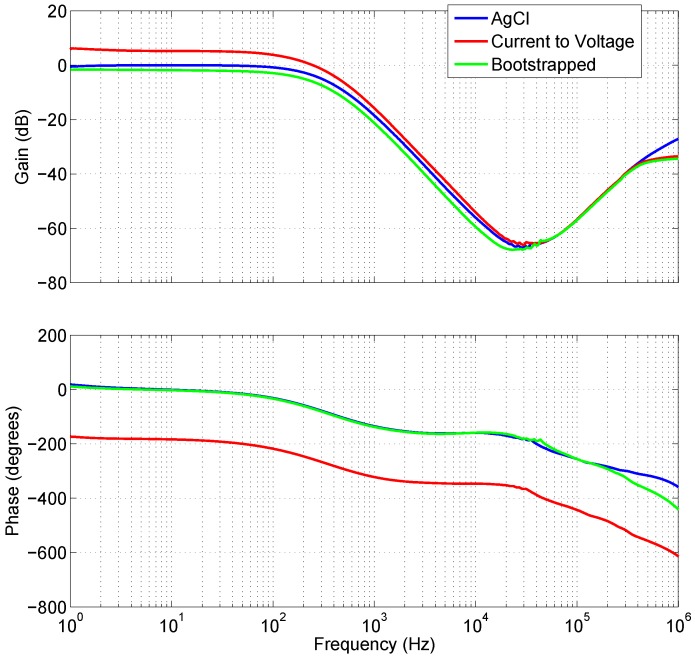
The gain and phase response of Ag/AgCl electrodes (blue) compared against the low impedance electrode (red) and high impedance (green).

Proven in experimental results, Figure 5 shows that the frequency and phase response of the AgCl electrodes plotted against the bootstrapped electrode are very nearly identical. Less than 0.3 dB gain separates the AgCl and Bootstrapped electrode, this is attributable to the depth of the insulator between the electrode and the skin, which lowers the coupling strength. The difference between the Bootstrapped and the current-to-voltage electrode results for the phase are 180°. This is expected due to the inverting configuration of the amplifier i.e., Equation (1) where the output is equal to the negative of the input current multiplied by the feedback resistance.

**Figure 6:**
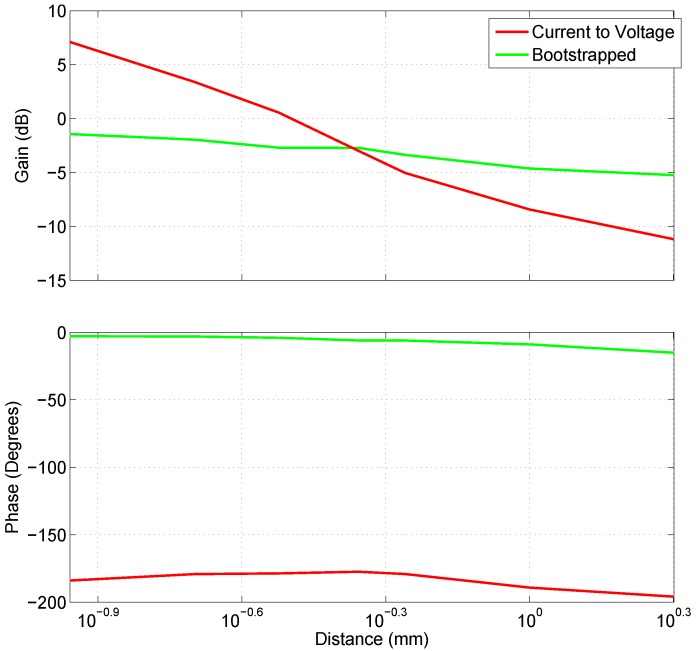
The gain at 10 Hz and phase at measured at 10 Hz with varying distances for the current-to-voltage converter (Red) and Bootstrapped electrode (Green).

Figure 6 illustrates the variation in gain and phase for the two types of capacitive electrodes at 10 Hz as the distance from the source is increased. While the variation in phase is similar and nondescript between the two, the drop in gain is steeper for the current-to-voltage converter configuration.

### 4.2. Subject Grounding

It was found throughout the experimentation that subject grounding plays an important part in keeping the environmental noise of the system to a minimum and can effect the amount of induced environmental noise substantially. The tests performed were achieved without any subject grounding and no galvanic contact when concerning the capacitive electrodes (electrical contact was necessary when comparing against AgCl electrodes but no grounding was used). Even ungrounded, it is still (without processing) possible to see all of the classical biological markers of the heartbeat waveform.

#### 4.2.1. Electrocardiograph Signals

For the acquisition of ECG signals, a differential measurement was used with electrodes placed thoracically, below the left and right pectoral muscles. This was achieved by recording ECG signals from male subjects’ chests using Ag/AgCl Medi-Trace Conductive ECG electrodes on one differential channel and capacitive electrodes over the subject’s clothes as topographically close to the Ag/AgCl electrodes as possible, connected to another channel of the data acquisition system. To further ascertain the fidelity of physiological recordings using the developed electrodes, we quantified a similarity between signals attained from them and those simultaneously recorded using Ag/AgCl electrodes. To assess this similarity, the magnitude squared coherence (MSC) was calculated by:

(9) $$C_{xy}\left(f\right)=\frac{|P_{xy}{\left(f\right)|}^{2}}{P_{xx}\left(f\right)P_{yy}\left(f\right)}$$

where:

$C_{xy}\left(f\right)$ = magnitude squared coherence estimate
$P_{xy}\left(f\right)$ = cross power spectral density of signals *x* and *y*
$P_{xx}\left(f\right)$ = power spectral density of signal *x*
$P_{yy}\left(f\right)$ = power spectral density of signal *y*

**Figure 7:**
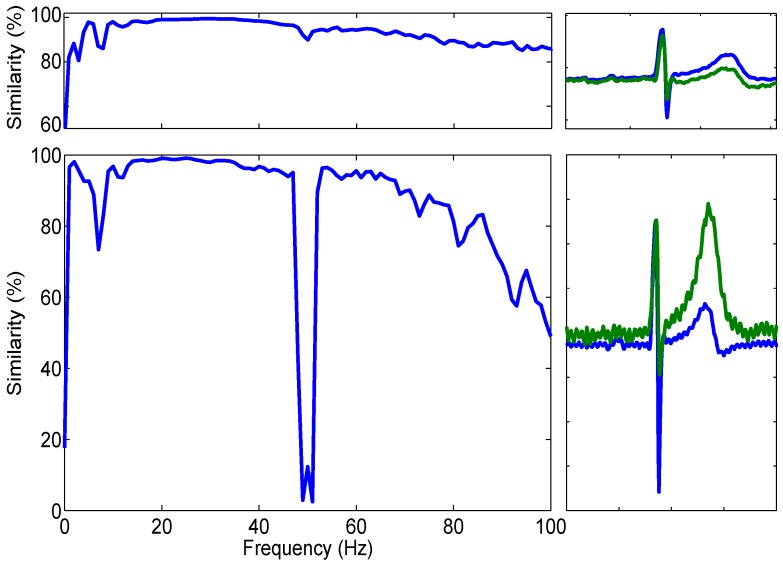
Left shows similarity plots of the capacitive electrode data, Bootstrapped electrode (**top**); and current-to-voltage converter (**bottom**). Right shows raw 1 s excerpts of the bio-potential recordings (blue Ag/Cl, green capacitive electrode).

This calculated how well *x*, the signal trace recorded from the Ag/AgCl electrodes, and *y*, the capacitive electrode trace, corresponded with each other for the given frequency range (0–100 Hz). The result is a number between 0–1 where 1 is absolute coherence and 0 is none; this result was multiplied by 100 to give a percentage. This is illustrated in the left hand plots of Figure 7.

Both plots of Figure 7 show anatomically correct ECG acquired from the bootstrapped electrode (upper similarity plot) and the current-to-voltage configuration (lower plot).

The results of these calculations are shown in Figure 7. Each plot was generated using 2 min of recorded ECG data split into one second windows and 500 ms of overlap. The signals were software filtered using a fourth-order Butterworth response low pass filter with a cut off frequency specified at 100 Hz to exclude any higher frequency information not crucial for the acquisition of ECG bio-potentials.

It is clear, in Figure 7, that the QRS complexes (the combination of three of the stereotypical graphical deflections seen on a ECG) of the AgCl heartbeat signal are coincidental with those reported by the active electrodes. The estimated MSC within the region of 15 Hz and 48 Hz between the AgCl and Capacitive electrode signals is 98.54% in the shown example and further figures are shown in Table 1 . It is also clear however, that while the P-wave is reported to be of similar size to the AgCl electrode, the T-wave is of a much higher amplitude. This high amplitude T-wave is responsible for the lower similarity value present at 8 Hz.

Due to the inverting nature of the current-to-voltage converter electrode configuration, it was necessary to invert the data around the mid-rail voltage before normalisation and comparison. While this illustrates the coincidence of the QRS complex, it also means that the 50 Hz interference detected (which was not inverted when it was not amplified through the differential amplifier) was then inverted. This inversion therefore is responsible for the large drop in similarity between 48 and 52 Hz.

#### 4.2.2. Electromyographic Signals

**Figure 8:**
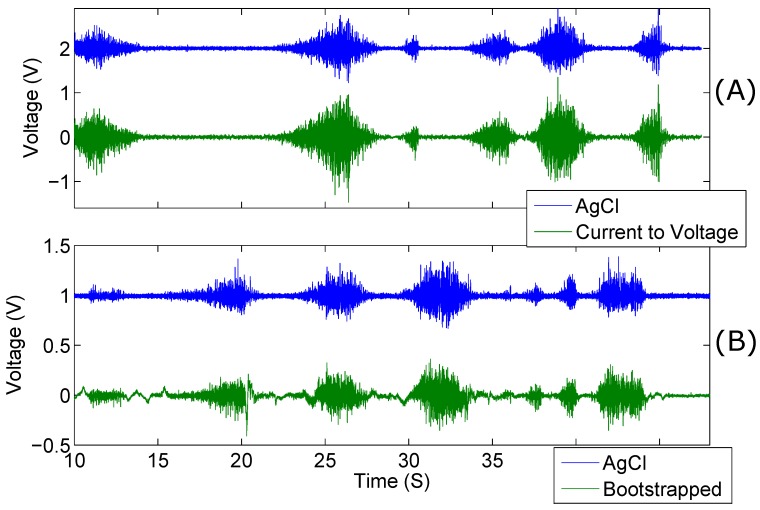
Comparison of electromyographic recordings taken from the right forearm of a male participant. AgCl vs. current-to-voltage converter electrode (**A**); and Agcl vs. bootstrapped electrode (**B**). The AgCl electrode recordings have been displaced for clarity.

Electromyographic recordings were taken differentially along the right forearm. Simultaneous recordings of both kinds of capacitive electrode were taken against AgCl electrodes placed. The results of one of the recording sessions is shown in Figure 8.

The gain of the AgCl electrode pair was set to 500 and the gain of the active electrodes was 250. Despite the lower gain and capacitive coupling of the active electrode pair, the amplitude is higher than that of the AgCl electrodes; this is evident in Figure 8. The data shown here is unprocessed with the exception of a 5 Hz high-pass filter applied to remove baseline drift. Despite this filtering, the Bootstrapped electrode configuration produced more low-frequency incoherences, as compared to the AgCl electrodes, than the current-to-voltage configuration. It is evident from the results shown in Table 2 that the low impedance electrodes, overall, have a higher mean MSC than the bootstrapped electrodes.

## 5. Discussion

The presented results show that capacitive electrodes can acquire bio-potential signals that rival traditional methods. The graphs in Figure 7 show that the coherence between simultaneously acquired frequency spectra of Ag/AgCl and both the low and high impedance configuration of electrodes is consistently above 90% and is above 99% in the region of 17–58 Hz.

The ability for the low impedance configuration to acquire ungrounded, subject, bio-potential signals without any special electrical considerations or equipment will be particularly useful for pervasive monitoring studies. The low preparation time in combination with a wireless transmission interface makes these electrodes ideal for pervasive monitoring applications e.g., early detection of cardiac events.

In recent work [22,23,24,25], phase relationships between signals originating from the cerebral cortex have been examined to obtain greater depths of information about neural and cognitive processes. It is with this in mind that the phase responses of the developed sensors have been closely examined, especially in cases where feedback and passive components could cause variation.

The size of the electrode was able to be minimised by choosing sufficiently small electronic packages with appropriate characteristics and circuit design techniques. Using the LMP7715 amplifier, the electrode size was able to be made as small as 15 mm in diameter, a significant decrease over other capacitive electrode designs.

In this paper, an inverting capacitive electrode design is presented and compared to a more traditional bootstrapped follower due to their differing properties, construction and ability to acquire bio-potential signals. While both designs are able to acquire and re-produce bio-potential data and, when directly compared with Ag/AgCl electrodes, produce high (minimum 90%) similarity, the low impedance electrodes capability of recording high quality EMG signals on ungrounded subjects makes them stand out, especially for ambulatory applications.

## 6. Conclusions

The capacitively coupled acquisition of bio-potentials holds the promise to significantly simplify the ability to acquire, store and manipulate bio-potential data, whilst providing a greatly reduced maintenance cycle and increasing patient comfort.

The implemented current-to-voltage electrodes have been demonstrated to be able to acquire bio-potential signals from a human body, which can acquire signals with a significant similarity to that of conductive wet electrodes, without electrical contact.

Coupled with advances in semi-conductor technology and wireless interfaces, this technology has potential to become ubiquitous in commercial industries. The development of devices that have no electrical contact with a subject are intrinsically safe and hence have no need for medical safety approval. This will make development of such devices significantly cheaper and may open attractive possibilities for casual application of brain computer interfaces and monitoring devices. Future developments will rely on acquiring bio-potential signals using a system composed of differing configurations of electrodes. This is with a view to take advantage of the current sensing ability of these electrodes and the voltage sensing ability of more traditional active electrodes.

## Figures and Tables

**Table 1:** Values of the mean of the MSC values between 15 and 48 Hz for capacitive ECG (cECG).

Subject	Mean MSC (current-to-voltage)	Mean MSC (Bootstrapped)
1	95.4%	96.8%
2	98.54%	97.55%
3	99.1%	98.34%

**Table 2:** Values of the mean of the MSC values obtained from contact acquired, Paynter filtered EMG data.

Subject	Mean MSC (current-to-voltage)	Mean MSC (bootstrapped)
1	99.2%	98.44%
2	98.8%	96.55%
3	99.5%	97.24%

## Abbreviations

The following abbreviations are used in this manuscript:

ECG	Electrocardiography
EMG	Electromyography
EEG	Electroencephalography
BCI	Brain Computer Interfacing
DRL	Driven Right Leg
PC	Personal Computer
TI	Texas Instruments
OPAMP	Operational Amplifier
PCB	Printed Circuit Boards
CMRR	Common-mode Rejection Ratio
A/D	Analogue-to-digital
DAQ	Data Acquisition System
SPI	Serial Peripheral Interface
